# Wiring the oncogenic circuitry: Pin1 unleashes mutant p53

**DOI:** 10.18632/oncotarget.329

**Published:** 2011-09-15

**Authors:** Marco Napoli, Javier E. Girardini, Silvano Piazza, Giannino Del Sal

**Affiliations:** ^1^Laboratorio Nazionale CIB (LNCIB), Area Science Park, 34149 Trieste, Italy; ^2^Dipartimento di Scienze della Vita, Università degli Studi di Trieste, 34127 Trieste, Italy; ^3^Instituto de Biologia Molecular y Celular de Rosario (IBR – CONICET), S2002LRK Rosario, Argentina

Unlike several tumor suppressor genes, whose inactivation is due to deletions or truncating mutations, *TP53* is most frequently hit by missense mutations in its DNA binding domain. Three are the functional consequences of these single amino acid substitutions: i) abrogation of tumor suppressor activities largely due to the inability to recognize wild-type p53 (wtp53) consensus sequences on DNA; ii) inhibition of the tumor suppressor function of the remaining wtp53 allele because of a dominant negative effect; iii) acquisition of new oncogenic properties, commonly described as mutant p53 (mutp53) gain of function, which can actively contribute to various aspects of tumor progression [1].

Intensive research in the last decade has definitively confirmed that mutp53 promotes the development of aggressive tumors, characterized by a metastatic phenotype and high levels of genomic instability [2-4]. Elegant *in vivo* studies have suggested that mutp53 requires additional alterations to exert its gain of function activities. Indeed, these properties were shown to be associated with mutp53 phosphorylation, suggesting that efficient mutp53 function may be triggered by oncogenic signaling, similarly to what occurs in the case of wtp53 [4, 5]. Very recently, we have unveiled a crucial link between cancer-related signaling and mutp53 oncogenic function: the prolyl isomerase Pin1, which transduces proline-directed signaling into mutp53 activation [5].

**Figure 1:**
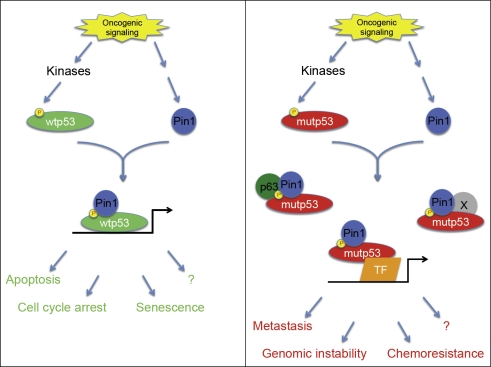
wtp53 versus mutp53 shows similar activation but opposite effects Upon oncogenic signaling, both wtp53 and mutp53 become activated by mechanisms involving several common kinases, such as p38, JNK and others, that modify the same residues. These phosphorylations allow Pin1 binding, which leads to full activation of both p53 proteins. Despite these similar steps, the downstream events associated to wtp53 or mutp53 are totally different. While the Pin1/wtp53 cooperation triggers tumor suppressor responses including apoptosis and cell cycle arrest, the Pin1/mutp53 axis promotes pro-oncogenic properties (such as cell migration and invasion, and possibly others like genomic instability, chemoresistance, etc.)

Our findings underline the similarities between wtp53 and mutp53 stimulation, although the biological outcomes of Pin1 binding and isomerization in either case are completely different (Figure 1). Indeed, we and others have previously established that Pin1 is essential for the proper activation of wtp53 function upon genotoxic stress. This enzyme was shown to promote both the dissociation of wtp53 from the E3 ubiquitin ligase Mdm2, thus increasing its stability, and the recruitment on target gene promoters contributing to specific tumor suppressor responses such as cell cycle arrest or apoptosis [6-10]. On the contrary, the cooperation between Pin1 and mutp53 gives rise to very opposite effects, since these proteins become integrated into a molecular axis supporting oncogenic mechanisms [5].

The existence of the Pin1/mutp53 axis was revealed in a Li-Fraumeni mouse model, where lack of Pin1 reduced tumorigenesis exclusively in mice expressing mutp53. In an *in vitro* model of cell transformation using primary fibroblasts derived from those animals, we identified Ras signaling as one of the pathways converging on mutp53 to increase phosphorylation and interaction with Pin1. At the same time, lack of Pin1 impaired the ability of mutp53 to promote anchorage-independent growth and tumorigenicity of H-Ras^V12^ transformed fibroblasts, thus indicating that oncogenic signaling *per se* cannot activate mutp53 unless Pin1 is present to bind mutp53 and unleash its gain of function properties.

In human breast cancer cells, activation of the Pin1/mutp53 axis fires oncogenic processes with deleterious consequences. In particular, these two proteins cooperate in enhancing cell migration and invasion by two independent but complementary mechanisms: the inhibition of the anti-metastatic factor p63 and the induction of a pro-aggressiveness transcriptional program. Both require mutp53 phosphorylation and interaction with Pin1 and result in a substantial reprogramming of gene expression that includes repression of tumor suppressor genes, comprising several p63 targets, as well as activation of genes promoting oncogenic processes. Among the genes induced by this axis, we have identified a group of 10 novel mutp53 direct target genes (that we defined as Pin1/mutp53 signature), which are regulated by the concerted action of these two proteins. Indeed, we demonstrated that Pin1 is required for efficient mutp53 recruitment on the promoters of this set of genes, suggesting that this prolyl isomerase could strengthen the complex between mutp53 and transcription factors tethering it on these promoters. It is also reasonable to believe that Pin1 might sustain expression of these genes by enhancing the ability of mutp53 to recruit transcriptional cofactors like the acetyl transferase p300, as Pin1 does to increase wtp53 transcriptional activity [9].

We confirmed the role of some of the 10 signature genes as downstream effectors of the Pin1/mutp53 axis, showing that they are directly involved in the promotion of migration and invasion. Accordingly, all these genes are likely to be involved in the progression toward a metastatic phenotype *in vivo*, since their high expression levels in tumors of breast cancer patients correlate with poor prognosis in terms of both reduced overall survival and shorter time to distant metastasis. Furthermore, in breast cancer data sets we have now found an intriguing association between the Pin1/mutp53 signature and tumor grade according to the Nottingham scale (unpublished data). Expression of the signature genes was found to be lower in G1 cases but high in G3 cases. In addition, it is also able to divide G2 cases into two subgroups: the G2-high-level-signature patients characterized by worse prognosis and the G2-low-level-signature patients having better prognosis. G2 tumors have an uncertain prognosis and represent the majority of cases [11], therefore, expression of the Pin1/mutant p53 signature genes could be very useful for proper clinical management of G2 patients.

The clinical relevance of the Pin1/mutp53 axis is further supported by our analysis of a cohort of breast cancer patients, in which we could establish that the prognostic value of p53 status is conditioned by Pin1 levels. The correlation between *TP53* mutation and poor prognosis has been pointed out several years ago, however, due to controversial data, the clinical use of *TP53* mutation as a prognostic marker was delayed [12]. Although some inconsistencies may result from the inaccuracy of evaluating *TP53* mutation indirectly, it is possible that *TP53* mutation becomes a powerful prognostic marker only when combined to the detection of other parameters, reflecting the fact that other alterations are required to fully activate or cooperate to oncogenic function of mutp53. Indeed, our data from breast cancer patients support this notion, since only in tumors expressing high Pin1 levels the presence of *TP53* missense mutations was associated to a worse clinical outcome with reduced overall survival and early development of metastases.

Our findings suggest that assembly of the Pin1/mutp53 axis may be a critical event that tips the balance toward tumor aggressiveness with important consequences on clinical management. In fact, according to Pin1 expression and *TP53* mutation or to the levels of the 10 signature genes, patients could be actually stratified in low- and high-risk groups. Our results not only give a significant contribution to the identification of cases not effectively responsive to the available therapies, but also suggest several potential strategies for the development of novel therapies to treat breast tumors or other epithelial cancers expressing mutp53 and high Pin1 levels, as for example preventing mutp53 phosphorylation and/or interaction with Pin1 as well as down-regulating the 10 signature genes.

Altogether our findings shed light on previously unknown details of the molecular mechanisms of tumor aggressiveness by revealing the crucial role of Pin1 in connecting oncogenic signaling with mutp53 gain of function. Nevertheless, several interesting issues worthy of further investigation remain. For instance, does Pin1 impact on other mutp53 associated processes, such as genomic instability and chemoresistance? Which are the mechanisms underlying transcriptional regulation by Pin1 and mutp53? Can Pin1 affect the prognostic value of *TP53* mutation in other kinds of tumors? Does high expression of the 10 signature genes correlate with poor prognosis also in those cases? Do these genes mediate other aspects of mutp53 gain of function? The answers to these and other questions will help to further elucidate mutp53-dependent oncogenic mechanisms and to provide the basis for novel therapeutic strategies.
